# A Multipurpose, High-Throughput Single-Nucleotide Polymorphism Chip for the Dengue and Yellow Fever Mosquito, *Aedes aegypti*

**DOI:** 10.1534/g3.114.016196

**Published:** 2015-02-26

**Authors:** Benjamin R. Evans, Andrea Gloria-Soria, Lin Hou, Carolyn McBride, Mariangela Bonizzoni, Hongyu Zhao, Jeffrey R. Powell

**Affiliations:** * Department of Ecology and Evolutionary Biology, Yale University, New Haven, Connecticut 06511; † Department of Biostatistics, Yale School of Public Health, New Haven, Connecticut 06520; ‡ Princeton Neuroscience Institute and Department of Ecology and Evolutionary Biology, Princeton University, Princeton, New Jersey 08540; § Department of Molecular Biology and Biochemistry, University of California Irvine, Irvine, California 92697

**Keywords:** genomics, population genetics, *Aedes aegypti*, SNP, vector biology

## Abstract

The dengue and yellow fever mosquito, *Aedes aegypti*, contributes significantly to global disease burden. Genetic study of *Aedes aegypti* is essential to understanding its evolutionary history, competence as a disease vector, and the effects and efficacy of vector control methods. The prevalence of repeats and transposable elements in the *Aedes aegypti* genome complicates marker development and makes genome-wide genetic study challenging. To overcome these challenges, we developed a high-throughput genotyping chip, Axiom_aegypti1. This chip screens for 50,000 single-nucleotide polymorphisms present in *Aedes aegypti* populations from around the world. The array currently used genotypes 96 samples simultaneously. To ensure that these markers satisfy assumptions commonly made in many genetic analyses, we tested for Mendelian inheritance and linkage disequilibrium in laboratory crosses and a wild population, respectively. We have validated more than 25,000 of these markers to date, and expect this number to increase with more sampling. We also present evidence of the chip’s efficacy in distinguishing populations throughout the world. The markers on this chip are ideal for applications ranging from population genetics to genome-wide association studies. This tool makes rapid, cost-effective, and comparable genotype data attainable to diverse sets of *Aedes aegypti* researchers, from those interested in potential range shifts due to climate change to those characterizing the genetic underpinnings of its competence to transmit disease.

Arthropod-borne diseases affect millions of people world-wide every year (World Health Organization 2014). Many of these diseases are best controlled by the elimination or suppression of their vector(s), because they have no effective vaccines or known cure. Historically, pesticides, habitat modification or destruction, and vector avoidance have played an important role in the local elimination or suppression of disease transmission via arthropod vectors. Additionally, there is burgeoning industry and scientific interest surrounding the development, release, and surveillance of genetically modified strains of these vectors and/or their endosymbionts as a means to minimize disease risk (Beaty 2000).

*Aedes aegypti*, is a major vector of dengue, Chikungunya, and yellow fever viruses. It is found in tropical and subtropical climates worldwide and is estimated to transmit as many as 528 million dengue infections per year (Bhatt *et al.* 2013). Together with *Aedes albopictus*, *Ae. aegypti* is responsible for the spread of Chikungunya virus from Africa to the Caribbean (Cassadou *et al.* 2014) directly threatening the mainland Americas (Charrel *et al.* 2014). Genetic study of *Ae. aegypti* is requisite for characterizing everything from its patterns of spread to genetic factors underlying its competence as a disease vector. Thus, there is dire need for a standardized and genome-wide set of markers to meet the demands of large-scale genetic studies in *Ae. aegypti*.

As holds true for any molecular genetics research, choosing an appropriate genotyping strategy is key to informative genetic studies in this system; however, this choice is accompanied by general and species-specific challenges. As is the case for many eukaryotes, whole-genome sequencing for routine genetic screening is cost-prohibitive and unnecessary in *Ae. aegypti*. Moreover, the structure, size, and content of the *Ae. aegypti* genome makes the collection, interpretation, and analysis of whole-genome data difficult. At least two thirds of the nuclear genome is composed of repeats, duplications, or transposable elements (Nene *et al.* 2007), and there are several nearly complete nuclear copies of the mitochondrial genome (Hlaing *et al.* 2009). Microsatellites, sometimes referred to as simple sequence repeats, or SSRs, are highly informative and mutable markers; yet, they require significant expertise and investment to develop. Even with access to the entire *Ae. aegypti* genome the yield was ~30 or fewer useable loci (Lovin *et al.* 2009; Brown *et al.* 2011). Microsatellites also are subject to human error in scoring and require special case-by-case attention in cross-study and cross-laboratory comparison, making their use as high-throughput markers difficult (Jones *et al.* 1997; Van Oosterhout *et al.* 2004; Pasqualotto *et al.* 2007). Single-nucleotide polymorphisms (SNPs) are information-poor when analyzed in small numbers, because they are usually chosen to be biallelic. Nevertheless, their simplicity and abundance allows one to compensate for per-marker information content by screening large numbers (Morin *et al.* 2004; Weller *et al.* 2006; Smouse 2010). Although bias attributed to the way the SNPs were attained does shift allele frequency spectra, with care this can also be corrected for (Nielsen *et al.* 2004). Restriction-site associated DNA sequencing, or RAD-seq (Hohenlohe *et al.* 2010; Peterson *et al.* 2012), recently has become a popular way to simultaneously identify and screen for SNPs across genomes, but data analysis is nontrivial, and there is still debate as to what is best practice for handling and interpreting missing data (Arnold *et al.* 2013; Huang and Knowles 2014).

Under these circumstances, and especially for this mosquito species, the development of a genotyping chip provides an excellent alternative for fast, high-throughput, and cost-effective screening of thousands to millions of SNPs. Ideally, markers included in a chip should have the following characteristics: 1) There should be as many markers as possible; 2) they should be present as a single copy in the genome; 3) they should be inherited in a Mendelian fashion, and 4) they should be distributed across known positions in the genome so that physical linkage can be distinguished from linkage disequilibrium due to other processes.

To this end, we developed a genotyping chip that contains probes for 50,000 SNP loci, distributed throughout the *Ae. aegypti* genome. Here, we validate a subset of the markers included on this chip, and show that these markers yield results consistent with previous studies of genetic variation in *Ae. aegypti*.

## Materials and Methods

### Mosquitoes used in double digest RAD-seq (ddRAD-seq)

*Ae. aegypti* were collected from 25 locations from around the world (Table 1) for SNP development and genotyping. Some of these population samples were used in a previous study (Brown *et al.* 2014), although the individuals are different. Mosquitoes were collected as eggs in the field and reared to adulthood, or were obtained as preserved larvae collected in the field. Genomic DNA was extracted for each individual mosquito using DNeasy extraction kits (QIAGEN).

**Table 1:** Sample population counts and sequencing data type

Location	RAD	ddRAD	RNA
Bijagos, Guinea-Bissau	8		
Bundibugyo, Uganda	8		Pooled
Cachoeiro, Brazil	8		
Cairns, Australia	8		
Chetumal Strain			Pooled
Dominica	8		
Hawaii, USA	8		
Houston, Texas, USA	8		
Kedougou, Senegal		3	
Mombassa, Kenya		12	
Kichwamba,Uganda	8		
Lunyo, Uganda		6	
Maraba, Brazil		3	
Natal, Brazil		3	
Pijijiapan, Mexico	8		
Puerto Rico, USA	8	6	
Rabai, Kenya (Indoor)	8		Pooled
Rabai, Kenya (Outdoor)	8		Pooled
Rayong, Thailand	8	6	
Rockefeller Strain			Pooled
Sedhiou, Senegal		3	
Tahiti, French Polynesia	8		
Tapachula, Chiapas, Mexico		6	
Vaca Key, FL, USA	8		
Yaoundé, Cameroon	8		

RAD, restriction-site associated DNA; ddRAD, double digest restriction-site associated DNA.

### RNA-seq marker development

Fastq data from Sequence Read Archive accession numbers SRP015697 and SRP035216 were aligned to the *Ae. aegypti* reference genome by TopHat (version 2.0.5). We removed reads generated from polymerase chain reaction duplicates with Picard v1.96, and reads with mapping quality lower than 30 were excluded for further analysis. Variants were called separately for each sample by GATK UnifiedGenotyper using default parameters only in regions from a list of genes compiled from literature associated with dengue competence and insecticide resistance (Supporting Information, Table S1). This decision was made partially due to computational restraints, but mostly because there was already an abundance of data to choose from in the analysis of the RAD-seq data. Variants were then filtered for depth of coverage (7×) and allele frequency (minor allele frequency ≥0.2). To avoid multiallelic sites, we calculated the percentage of reads with either reference allele or the called alternative allele in the total number of reads covering the corresponding site and those with less than 0.85 were left out for further analysis. These potential sites were further filtered for 35 bp of invariant sequence on each side to allow for probe design.

### RAD sequencing and marker development

RAD-seq DNA samples were sent to Floragenex in Eugene, Oregon, for library preparation. ddRAD DNA samples were prepared at Yale using the protocol described in the ddRAD manuscript (Peterson *et al.* 2012). Genomic DNA was digested with enzymes *MluC*I and *Nla*III (New England BioLabs), barcoded with oligos described in Peterson *et al.* (2012), and fragments were size-selected to be ~195 bp ± 20 bp of genomic DNA with a BluePippin (Sage Science). This size was chosen to maximize the markers recovered per-individual and increase the chances of finding SNPs with 35 bp of known flanking invariant sequence. Two 24-individual ddRAD libraries were sequenced on two lanes on an Illumina Hisequation 2000 at the Yale Center for Genome Analysis. All RAD sequence data were mapped to the *Ae. aegypti* reference genome (Nene *et al.* 2007) (AaegL1) using Bowtie2 (Langmead and Salzberg 2012). Roughly 693,000 variants were identified using GATK2 (McKenna *et al.* 2010) Haplotype Caller using default settings, then filtered for depth of coverage (minimum 7x), quality (minimum variant phred score 25), missing data (maximum missing 20%), and 35 bp of invariant sequence on each side to allow for probe design. These variants, combined with those identified in the RAD-seq data described in Brown *et al.* 2014), produced a set of 139,654 potential probes. These probes were then scored using the Affymetrix SNP chip design pipeline, taking into account which probe pairs will perform well based on thermodynamics, self-hybridization, and copy number present in the reference genome. The 50,000 best rated sets of probes were chosen for final printing of the chip, Axiom_aegypti1. For a list of probe sequences and genome positions, see Table S2.

### Positional bias

We measured Pearson’s r, using the scipy.stats v 0.14 function pearsonr, between pairwise SNP distance and supercontig length to test whether there is significant positional bias between SNPs across the genome.

### SNP genotyping and quality control

Genomic DNA was sent to The Functional Genomics Core at University of North Carolina, Chapel Hill in batches of 95 samples with one negative control and genotyped using manufacturer protocols. We analyzed Axiom_aegypti1 data using Genotyping Console v4.2 using default parameters outlined as best practice for non-human samples, and the R package SNPolisher v1.4 (Affymetrix, Santa Clara, CA) with default parameters, except with the call threshold set to 95%, to generate and post-process genotype calls. We genotyped a total of 160 wild and 101 lab-reared mosquitoes for this study. Full probe-by-probe results can be found in Table S3.

### Mendelian inheritance

We generated single pair matings from 5 laboratory colonies (Table S4), which generated 27 cohorts of F_1_ offspring. All parents were genotyped using Axiom_aegypti1. We chose 32, 31, and 32 individual F1 offspring from crosses 5, 16, and 25, respectively. These crosses were chosen for genotyping so that they maximized the number of SNPs where the parental genotypes were heterozygous in one parent and homozygous in the other. This allowed for maximal power in testing of Mendelian inheritance across markers. We performed χ^2^ tests comparing expected and observed genotype frequencies in each of the three cohorts separately, combining *P* values where possible using Fisher’s method. We were able to test a total of 16,111 SNP loci in at least one cohort. These *P* values were then sequential Bonferroni-Holm corrected.

### Linkage disequilibrium

We carried out pairwise genotypic r^2^ tests on SNPs not eliminated in quality control or Mendelian tests. We measured linkage disequilibrium as implemented in plink v1.07 (Purcell *et al.* 2007), with no distance or r^2^ filters, on 40 individuals collected from Jacobina, Bahia, Brazil. We generated a log-scaled two-dimensional histogram of r^2^ values *vs.* distance using the python package matplotlib v1.4.0. Locally weighted scatterplot smoothing curves were generated with python package statsmodels v0.5.0 for r^2^ greater than or equal to 0.2 and all r^2^ values.

### Principal components analysis (PCA) and geographic assignment

We used a scaled matrix of genotype frequencies for wild mosquito samples for a PCA as implemented in the python package scikit-learn (Pedregosa and Varoquaux 2011). To avoid results biased by oversampling, we randomly subsampled the population of 40 individuals down to 12 (Table 2).

**Table 2:** Sample counts and origins for Axiom_aegypti1 genotyping

Population	Sample Count
Goudiry, Tambacounda, Senegal	12
Lunyo, Entebbe, Uganda	12
Sedhiou, Sedhiou, Senegal	12
Cairns, Queensland, Australia	12
Tahiti, French Polynesia	12
Rayong, Thailand	11
Jacobina, Bahia, Brazil	40 (12)
Tapachula, Chiapas, Mexico	12
Hawaii, Hawaii, USA	6
Key West, Florida, USA	11
Patillas, Puerto Rico, USA	12
Houston, Texas, USA	8
Total	160 (132)

The numbers in parentheses are those used for principal component analysis (Figure 2).

## Results

**Figure 1:**
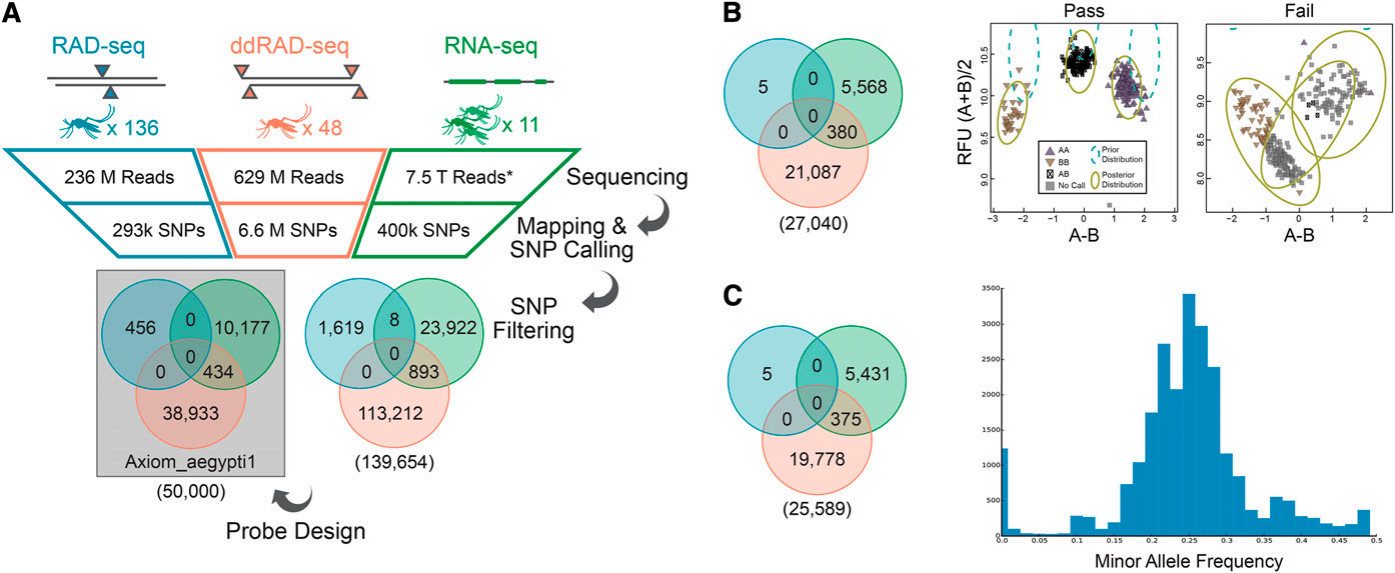
Summary of the validation pipeline. (A) The number of samples, sequencing reads, and SNPs that were filtered to the final 50,000 SNPs for Axiom_aegypti1. Colors of each data type are maintained throughout all panels, numbers in parentheses are totals of Venn diagrams. (B) The number of SNPs retained after quality control. Raw chip data were base-called with Affymetrix software. SNPs that were not called in 95% or more of samples were marked as failed for downstream analysis. Pass and Fail are examples of a failed and passes SNP call. The Y axis is the average florescence from each color channel (there are two for each SNP position: A and B). The X-axis is the difference in intensity between color channels. Each point on the plots represents an individual sample. Pure, high-intensity signals in one color or another are indicative of a homozygote, while a sample with equal parts of both colors is indicative of a heterozygote. (C) The number of SNPs retained after testing for Mendelian Inheritance. The histogram shows the distribution of minor allele frequency in 95 F_1_ offspring from three single parent crosses where one parent was homozygous and the other heterozygous. SNP, single-nucleotide polymorphism.

**Figure 2:**
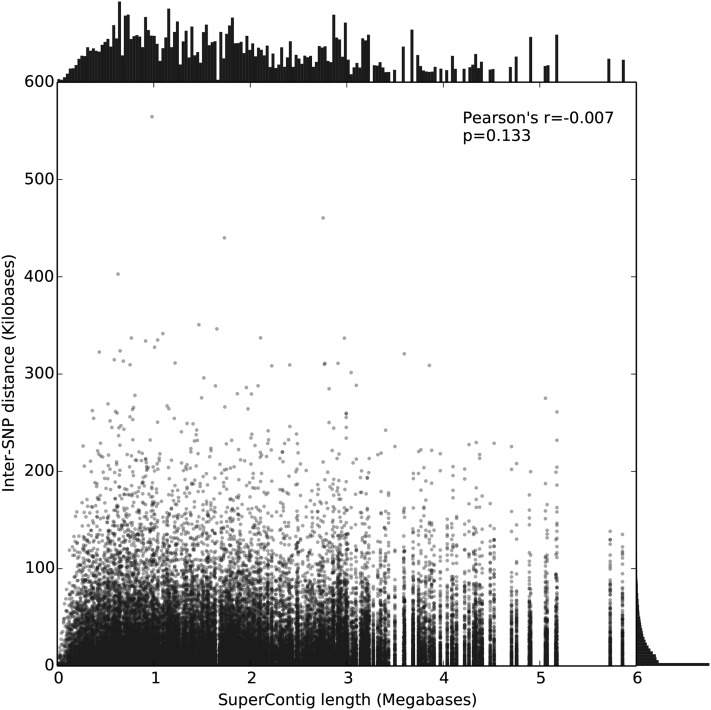
Scatter plot of inter-SNP distances *vs.* the length of the supercontig on which the pair are located. The mean intermarker distance is 24,741 bp. The upper and right-hand edges are univariate distributions of the sizes and distances, respectively. Pearson product-moment correlation coefficient for distance *vs.* length of supercontig is −0.007, with a *P* value of 0.133. SNP, single-nucleotide polymorphism.

**Figure 3:**
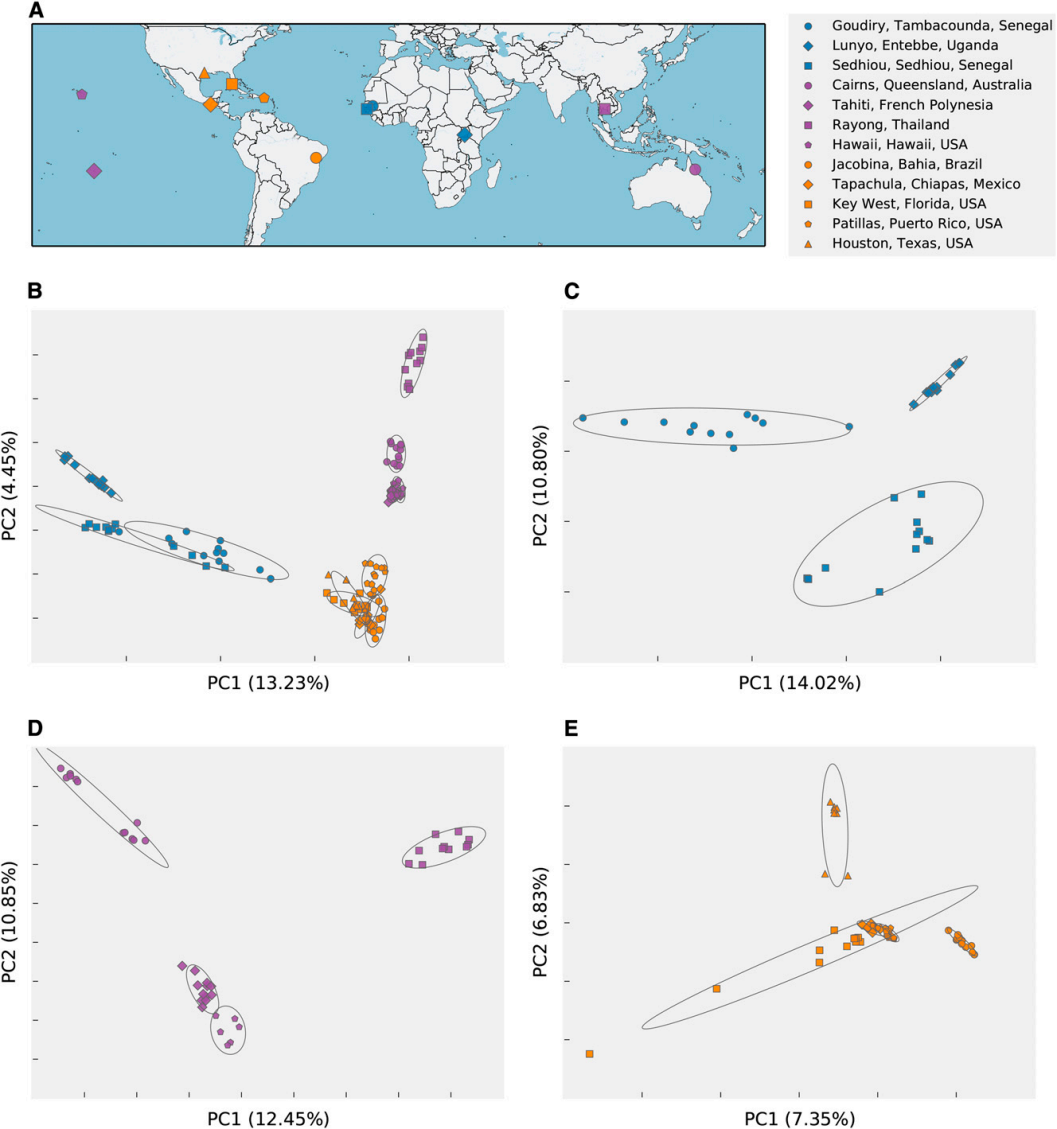
Geographic origin and patterns of genetic variation observed in wild samples genotyped with axiom_aegypti1. (A) Geographical origin of sample populations genotyped. Colors signify major global divisions: Blue indicates Africa; purple indicates Asia and the Pacific; and orange indicates the Americas. Symbols are used to further differentiate populations. Legend applies for entire figure. (B−E) Principal component analysis of genotypic variance in all populations (B), African populations (C), Asian and Pacific populations (D), and American populations (E).

To develop the SNP markers for this chip, we mined previous RAD-seq generated SNP data (Brown *et al.* 2014), RNA-seq data (McBride *et al.* 2014; Bonizzoni *et al.* 2012), and newly generated double-digest RAD-seq (Peterson *et al.* 2012) representative of populations of *Ae. aegypti* from around the world (Table 1). In the RNA-seq data we focused specifically on genes identified in a literature search as correlated or functionally related to dengue competence and insecticide resistance (Table S1). The rest of the SNPs were randomly distributed throughout the genome. After quality filtering the SNP data, we used the highest scoring 50,000 SNP markers as ranked for expected performance on an Affymetrix Axiom MyDesign array (Figure 1A), which we named Axiom_aegypti1. There was little overlap between RAD-seq datasets, as expected when different digestion enzymes are used. The average genomic distance between SNPs on the chip is 24,741 bp, with at least one SNP on 1950 supercontigs. This set of supercontigs represents 96.95% of the total published genomic sequence (Nene *et al.* 2007) and constitutes 116 of the 120 supercontigs placed on the 3 *Ae. aegypti* chromosomes in a recent florescence *in situ* hybridization study (Timoshevskiy *et al.* 2013) (see Table S2 for full description of probes). There is no significant correlation between supercontig length and inter-SNP distance, which suggests that there is little if any bias in SNP position relative to others across the genome (Pearson’s r = −0.007, *P* = 0.133, Figure 2). The SNPs on the chip were further tested for array performance by genotyping 160 wild individuals from different populations (Table 2 and Figure 3A). A total of 10,183 SNPs showed low array performance, 7969 SNPs had evidence of a third allele or possible duplication event, and 4808 SNPs were monomorphic in populations genotyped (Figure 1B, Table 3, and Table S3). We then tested the remaining 27,040 SNPs for a Mendelian pattern of inheritance by using the chip to genotype three F1 full-sibling cohorts and their parents. We found 1451 SNPs to be significantly deviant from what is expected under a Mendelian genetic model of inheritance (Figure 1C, Table S4 and Table S5). This could either be due to true departure from a model of Mendelian inheritance, or due to a genotyping error in the parents. These positions were excluded, leaving 25,589 SNPs for further analysis.

**Table 3:** SNP Q/C based on 160 individual genotypes, 12 populations

SNP Classification	SNP Count	Percent Total
All possible genotypes	21,247	42.5%
No minor allele homozygotes	5793	11.6%
Monomorphic	4808	9.6%
>2 alleles present	7969	15.9%
Call rate below 95% threshold	1077	2.2%
Low quality	9106	18.2%

Q/C, Quality Control

**Figure 4:**
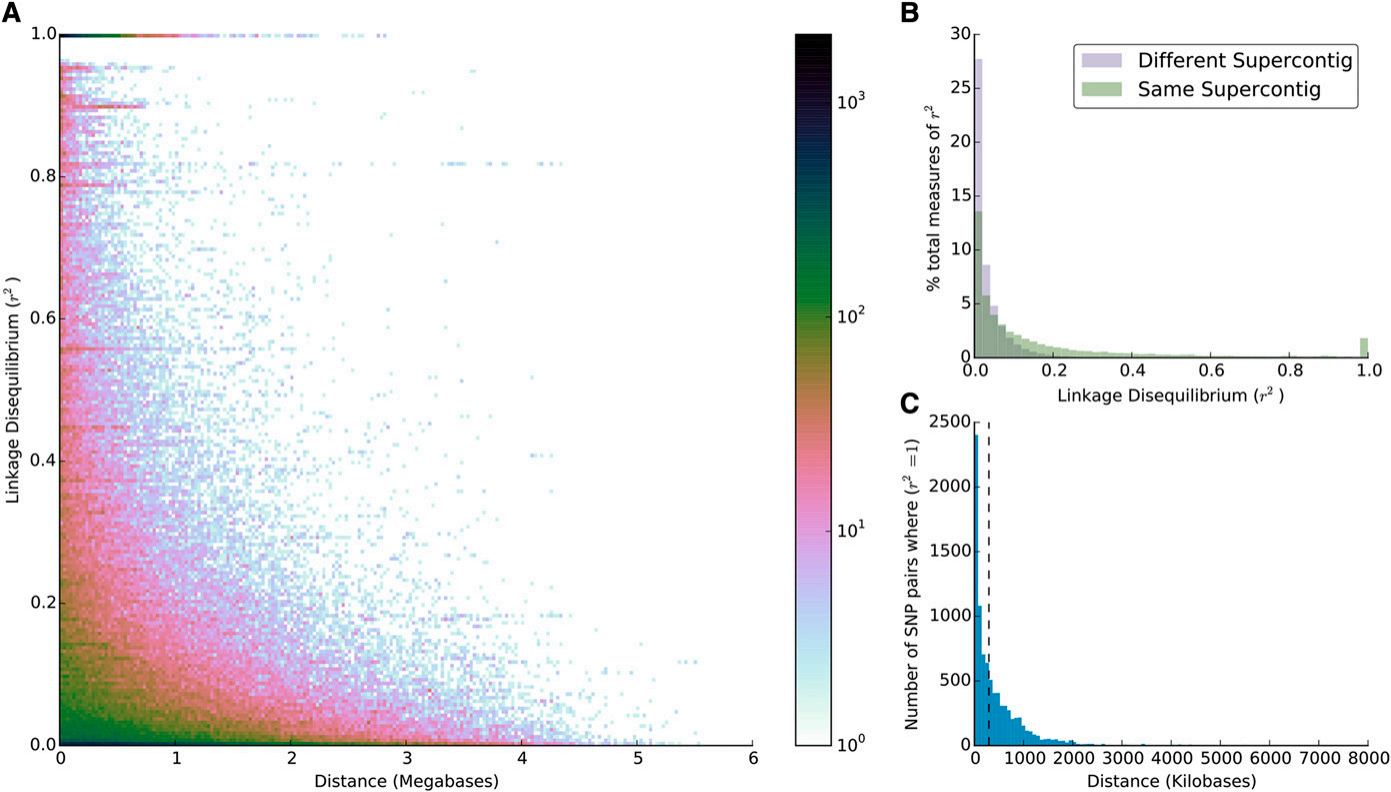
(A) A two-dimensional histogram with bins of 200 bp of pairwise measures of linkage, as measured by r^2^, for SNPs on the same supercontig. A given point’s color on the plot indicates the log-quantity of r^2^ values found between SNPs that are the same distance apart. The average value of r^2^ for this plot is 0.1797. (B) Relative distributions of r^2^ values for SNP pairs on the same (green) and different (blue) supercontigs. (C) Distribution of the number of SNP pairs on the same supercontig where r^2^ = 1 and the distance between them. A dashed line indicates the median distance of the distribution (147,669 bp).

We then examined pair-wise linkage disequilibrium, as measured by r^2^, in a South American population for which we had the highest sample size (n = 40). The global mean r^2^ value is 0.0348. The averages for SNP pairs on the same and different supercontigs are 0.1797 and 0.0345, respectively. When only SNP pairs with r^2^ values of 1 on the same supercontig are examined by themselves, 50% of these pairs are within a distance of ~148kb of one another (Figure 4C). We measured relatedness (as measured by the unadjusted Ajk statistic (Yang *et al.* 2010)) and tested for departures from Hardy Weinberg equilibrium in these SNPs. We found 3 individuals that may be related to one another (Figure S1 and Table S6), and less than 1% (204 SNP loci) that are out of Hardy Weinberg equilibrium after multiple test correction, α = 0.05 (Table S7).

Next we performed principal components analysis on the wild individuals to examine variation in natural populations. Strong geographic signal is evident in the first two principal components when examining all populations together (Figure 2B), as found in previous studies (Brown *et al.* 2014; Rašić *et al.* 2014). When analyzed separately (Figure 2, C−E), populations from Africa, Asia and Pacific Islands, and the Americas are clearly diagnosable at resolution not seen previously (Brown *et al.* 2011, 2014).

## Discussion

Here we describe the development of a SNP chip for *Ae. aegypti* and test a set of SNP markers useful in genotypic analysis of natural populations. This robust set of SNPs do not suffer from the difficulties inherent to the development and analysis of microsatellites (Chambers *et al.* 2007; Brown *et al.* 2011) or genotype-by-sequencing markers (Rašić *et al.* 2014). There are at least ~25,000 useful markers on the chip, named Axiom_aegypti1, that are Mendelian in nature and distributed throughout the *Ae. aegypti* genome.

With additional geographic sampling and more substantial population study, this preliminary and conservatively filtered set of SNPs will grow. Future results will benefit from the Bayesian base-calling algorithms employed in the genotyping pipeline, increasing the number of high-confidence SNPs per sample as well as the number of SNPs that pass quality control. Furthermore, although 4800 probes were monomorphic and therefore uninformative in our samples, some of these may prove polymorphic in future populations samples.

As more results are generated, we encourage their archival in public databases so they may be mirrored in Vectorbase (Megy *et al.* 2012). We are hopeful that this database will grow rapidly as data generation using this platform is fast, on the order of a few days. Using such an open framework will yield data that are easily combined, shared across research groups, and visualized, further lowering the barrier to synthesis and analysis. Researchers now have the capacity to rapidly generate and synthesize results to address questions that were previously recalcitrant or unanswerable in this system.

A database of genetic variation for *Ae. aegypti* is a valuable tool, especially in the face of the spread and increase in incidence of dengue and Chikungunya viruses. Cataloging genetic variation with Axiom_aegypti1 will be crucial to tracking and better understanding of the patterns of movement of *Ae. aegypti* at fine geographic and temporal scales. Refining current hypotheses on the evolutionary origins of *Ae. aegypti* (Moore *et al.* 2013; Powell and Tabachnick 2013; Brown *et al.* 2014), will improve understanding of current patterns of invasion and other questions in invasion biology and domestication. In addition to basic science, this database will be central to better understanding of the genetic underpinnings of genotypic factors in *Ae. aegypti* relevant to human health.

A more immediate evolutionary application of these markers is exploring how population structure and genetic differentiation affect the interface between wild populations and genetically modified strains being released to fight *Ae. aegypti* borne illness. This can, in turn, inform control strategies *e.g.*, timing of releases, measurement of program efficacy, etc. During the release of genetically modified organisms, detecting any level of introgression can be valuable to assess the spread of undesired traits into wild populations, to reduce public concerns, or to evaluate the effectiveness of different release strategies. Screens for single locus diagnostic alleles are likely to miss incipient introgression or hybridization where recombination has eliminated the gene that is being screened. Therefore, the ability to measure these genetic parameters at the scale of the genome provides a great asset to release programs employing genetically modified organisms. Various genetic control strategies, both currently being deployed and under development, are reviewed by McGraw and O’Neill (McGraw and O’Neill 2013). Genetic data in the face of these strategies can also help to assess the effects of a release in a more holistic evolutionary and ecological framework (David *et al.* 2013). Ultimately, the field application of these still nascent technologies demands rigorous and independent analysis of their effects and efficacy.

The identification of genetic backgrounds underlying various factors immediately relevant to human health will also benefit from using this tool. Laboratory study of recently caught wild populations with varying levels of competence to transmit virus or feeding behavior can be screened for genotypes that are especially susceptible or refractory to such phenotypes using GWAS as has been done in other systems(Welter *et al.* 2014). Given the average distance between SNPs on the Axiom_aegypti1 chip, the observed linkage disequilibrium (Figure 4A), and the fact that the *Ae. aegypti* genome constitutes ~200 cM (Severson *et al.* 2002; Timoshevskiy *et al.* 2013), this chip will also prove useful in quantitative trait locus mapping and genome-wide association studies; there are at least ~127 markers per centimorgan. In contrast to *Anopheles gambiae*, where linkage disequilibrium breaks down at distances as small as 200 bp (Harris *et al.* 2010; Weetman *et al.* 2010), these markers should prove informative to traits of interest at greater genomic distances in the larger genome of *Ae. aegypti* (Black Iv *et al.* 2008). Although the average r^2^ we measured in one South American population is globally low, there are good indications of many useful genome-wide association study markers (Figure 4). Larger-scale study is needed to further characterize linkage disequilibrium these markers in multiple populations from around the world. Overall, this tool shows promise to advance the genetic study of *Ae. aegypti* significantly while allowing those without extensive genomics backgrounds to participate and drive progress.

### Data availability

The Axiom_aegypti1 chip is available from Affymetrix in 96 sample Axiom array format. Individual-level data on the samples included in this study are available in European Molecular Biology Laboratory−European-Bioinformatics Institute BioSamples group SAMEG188691, and their genotyping information is mirrored in VectorBase (http://www.vectorbase.org).

## References

[bib1] Arnold B. Corbett-Detig R. B. Hartl D. Bomblies K., 2013 RADseq underestimates diversity and introduces genealogical biases due to nonrandom haplotype sampling. Mol. Ecol. 22: 3179–3190. 23551379 10.1111/mec.12276

[bib2] Beaty B. J., 2000 Genetic manipulation of vectors: a potential novel approach for control of vector-borne diseases. Proc. Natl. Acad. Sci. USA 97: 10295–10297. 10984525 10.1073/pnas.97.19.10295 PMC34037

[bib3] Bhatt S. Gething P. W. Brady O. J. Messina J. P. Farlow A. W., 2013 The global distribution and burden of dengue. Nature 496: 504–507. 23563266 10.1038/nature12060 PMC3651993

[bib4] Black W. IV Gorrochetegui-Escalante N. Randle N. Donnelly M., 2008 The yin and yang of linkage disequilibrium: mapping of genes and nucleotides conferring insecticide resistance in insect disease vectors, pp. 71–83 in *Transgenesis and the Management of Vector-Borne Disease SE - 6*, *Advances in Experimental Medicine and Biology*, edited by Aksoy S. Springer, New York. 10.1007/978-0-387-78225-6_6 18510015

[bib5] Bonizzoni M. Dunn W. A. Campbell C. L. Olson K. E. Marinotti O., 2012 Complex modulation of the *Aedes aegypti* transcriptome in response to dengue virus infection. PLoS One 7: e50512. 23209765 10.1371/journal.pone.0050512 PMC3507784

[bib6] Brown J. E. McBride C. S. Johnson P. Ritchie S. Paupy C., 2011 Worldwide patterns of genetic differentiation imply multiple “domestications” of *Aedes aegypti*, a major vector of human diseases. Proc. Biol. Sci. 278: 2446–2454. 21227970 10.1098/rspb.2010.2469 PMC3125627

[bib7] Brown J. E. Evans B. R. Zheng W. Obas V. Barrera-Martinez L., 2014 Human impacts have shaped historical and recent evolution in *Aedes aegypti*, the dengue and yellow fever mosquito. Evolution 68: 514–525. 24111703 10.1111/evo.12281 PMC3946797

[bib8] Cassadou S. Boucau S. Petit-Sinturel M. Huc P. Leparc-Goffart I., 2014 Emergence of chikungunya fever on the French side of Saint Martin island, October to December 2013. Euro Surveill. 19: 1–4. 10.2807/1560-7917.es2014.19.13.20752 24721536

[bib9] Chambers E. W. Meece J. K. McGowan J. A. Lovin D. D. Hemme R. R., 2007 Microsatellite isolation and linkage group identification in the yellow fever mosquito *Aedes aegypti*. J. Hered. 98: 202–210. 17420178 10.1093/jhered/esm015

[bib10] Charrel R. Leparc-Goffart I. Gallian P. de Lamballerie X., 2014 Globalization of Chikungunya: 10 years to invade the world. Clin. Microbiol. Infect. 20: 662–663. 24889861 10.1111/1469-0691.12694 PMC7128442

[bib11] David A. Kaser J. Morey A., 2013 Release of genetically engineered insects: a framework to identify potential ecological effects. Ecol. Evol. 3: 4000–4015. 24198955 10.1002/ece3.737 PMC3810890

[bib12] Harris C. Rousset F. Morlais I. Fontenille D. Cohuet A., 2010 Low linkage disequilibrium in wild *Anopheles gambiae* s.l. populations. BMC Genet. 11: 81. 20843306 10.1186/1471-2156-11-81 PMC2949739

[bib13] Hlaing T. Tun-Lin W. Somboon P. Socheat D. Setha T., 2009 Mitochondrial pseudogenes in the nuclear genome of *Aedes aegypti* mosquitoes: implications for past and future population genetic studies. BMC Genet. 10: 11. 19267896 10.1186/1471-2156-10-11 PMC2660364

[bib14] Hohenlohe P. Bassham S. Etter P., 2010 Population genomics of parallel adaptation in threespine stickleback using sequenced RAD tags. PLoS Genet. 6: e1000862. 20195501 10.1371/journal.pgen.1000862 PMC2829049

[bib15] Huang H. Knowles L. L., 2014 Unforeseen consequences of excluding missing data from next-generation sequences: simulation study of RAD sequences. Syst. Biol. 10.1093/sysbio/syu046. 10.1093/sysbio/syu046 24996413

[bib16] Jones C. J. Edwards K. J. Castaglione S. Winfield M. O. Sala F., 1997 Reproducibility testing of RAPD, AFLP and SSR markers in plants by a network of European laboratories. Mol. Breed. 3: 381–390.

[bib17] Langmead B. Salzberg S. L., 2012 Fast gapped-read alignment with Bowtie 2. Nat. Methods 9: 357–359. 22388286 10.1038/nmeth.1923 PMC3322381

[bib18] Lovin D. D. Washington K. O. DeBruyn B. Hemme R. R. Mori A., 2009 Genome-based polymorphic microsatellite development and validation in the mosquito *Aedes aegypti* and application to population genetics in Haiti. BMC Genomics 10: 590. 20003193 10.1186/1471-2164-10-590 PMC3087561

[bib19] McBride C. S. Baier O. F. Aman J. Lutomiah R. Sang R. I., 2014 Evolution of mosquito preference for humans linked to an odorant receptor. Nature 515: 222–227. 25391959 10.1038/nature13964 PMC4286346

[bib20] McGraw E. A O’Neill S. L., 2013 Beyond insecticides: new thinking on an ancient problem. Nat. Rev. Microbiol. 11: 181–193. 23411863 10.1038/nrmicro2968

[bib21] McKenna A. Hanna M. Banks E. Sivachenko A. Cibulskis K., 2010 The Genome Analysis Toolkit: a MapReduce framework for analyzing next-generation DNA sequencing data. Genome Res. 20: 1297–1303. 20644199 10.1101/gr.107524.110 PMC2928508

[bib22] Megy K. Emrich S. J. Lawson D. Campbell D. Dialynas E., 2012 VectorBase: improvements to a bioinformatics resource for invertebrate vector genomics. Nucleic Acids Res. 40: D729–D734. 22135296 10.1093/nar/gkr1089 PMC3245112

[bib23] Moore M. Sylla M. Goss L. Burugu M. W. Sang R., 2013 Dual African origins of global *Aedes aegypti* s.l. populations revealed by mitochondrial DNA. PLoS Negl. Trop. Dis. 7: e2175. 23638196 10.1371/journal.pntd.0002175 PMC3630099

[bib24] Morin P. a. Luikart G. Wayne R. K., 2004 SNPs in ecology, evolution and conservation. Trends Ecol. Evol. 19: 208–216.

[bib25] Nene V. Wortman J. R. Lawson D. Haas B. Kodira C., 2007 Genome sequence of *Aedes aegypti*, a major arbovirus vector. Science 316: 1718–1723. 17510324 10.1126/science.1138878 PMC2868357

[bib26] Nielsen R. Hubisz M. J. Clark A. G., 2004 Reconstituting the frequency spectrum of ascertained single-nucleotide polymorphism data. Genetics 168: 2373–2382. 15371362 10.1534/genetics.104.031039 PMC1448751

[bib27] Pasqualotto A. C. Denning D. W. Anderson M. J., 2007 A cautionary tale: lack of consistency in allele sizes between two laboratories for a published multilocus microsatellite typing system. J. Clin. Microbiol. 45: 522–528. 17166958 10.1128/JCM.02136-06 PMC1829014

[bib28] Pedregosa F. Varoquaux G., 2011 Scikit-learn: machine learning in Python. J. Mach. Learn. Res. 12: 2825–2830.

[bib29] Peterson B. K. Weber J. N. Kay E. H. Fisher H. S. Hoekstra H. E., 2012 Double digest RADseq: an inexpensive method for de novo SNP discovery and genotyping in model and non-model species. PLoS One 7: e37135. 22675423 10.1371/journal.pone.0037135 PMC3365034

[bib30] Powell J. R. Tabachnick W. J., 2013 History of domestication and spread of Aedes aegypti—a review. Mem. Inst. Oswaldo Cruz 108(Suppl): 11–17. 24473798 10.1590/0074-0276130395 PMC4109175

[bib31] Purcell S. Neale B. Todd-Brown K. Thomas L. Ferreira M. A. R., 2007 PLINK: a tool set for whole-genome association and population-based linkage analyses. Am. J. Hum. Genet. 81: 559–575. 17701901 10.1086/519795 PMC1950838

[bib32] Rašić G. Filipović I. Weeks A. R. Hoffmann A. A., 2014 Genome-wide SNPs lead to strong signals of geographic structure and relatedness patterns in the major arbovirus vector, *Aedes aegypti*. BMC Genomics 15: 275. 24726019 10.1186/1471-2164-15-275 PMC4023594

[bib33] Severson D. W. Meece J. K. Lovin D. D. Saha G. Morlais I., 2002 Linkage map organization of expressed sequence tags and sequence tagged sites in the mosquito, *Aedes aegypti*. Insect Mol. Biol. 11: 371–378. 12144703 10.1046/j.1365-2583.2002.00347.x

[bib34] Smouse P. E., 2010 How many SNPs are enough? Mol. Ecol. 19: 1265–1266. 20456228 10.1111/j.1365-294X.2010.04555.x

[bib35] Timoshevskiy V. A. Severson D. W. Debruyn B. S. Black W. C. Sharakhov I. V., 2013 An integrated linkage, chromosome, and genome map for the yellow fever mosquito *Aedes aegypti*. PLoS Negl. Trop. Dis. 7: e2052. 23459230 10.1371/journal.pntd.0002052 PMC3573077

[bib36] Van Oosterhout C. Hutchinson W. F. Wills D. P. M. Shipley P., 2004 Micro-checker: software for identifying and correcting genotyping errors in microsatellite data. Mol. Ecol. Notes 4: 535–538.

[bib37] Weetman D. Wilding C. S. Steen K. Morgan J. C. Simard F., 2010 Association mapping of insecticide resistance in wild *Anopheles gambiae* populations: major variants identified in a low-linkage disequilbrium genome. PLoS One 5: e13140. 20976111 10.1371/journal.pone.0013140 PMC2956759

[bib38] Weller J. I. Seroussi E. Ron M., 2006 Estimation of the number of genetic markers required for individual animal identification accounting for genotyping errors. Anim. Genet. 37: 387–389. 16879353 10.1111/j.1365-2052.2006.01455.x

[bib39] Welter D. MacArthur J. Morales J. Burdett T. Hall P., 2014 The NHGRI GWAS Catalog, a curated resource of SNP-trait associations. Nucleic Acids Res. 42: D1001–D1006. 24316577 10.1093/nar/gkt1229 PMC3965119

[bib40] World Health Organization, 2014 Vector-Borne diseases. Fact sheet N°387. Available at: http://www.who.int/mediacentre/factsheets/fs387/en/. Accessed: March 5, 2015.

[bib41] Yang J. Benyamin B. McEvoy B. P. Gordon S. Henders A. K., 2010 Common SNPs explain a large proportion of the heritability for human height. Nat. Genet. 42: 565–569. 20562875 10.1038/ng.608 PMC3232052

